# Toxicological evaluation of standardized *Kaempferia parviflora* extract: Sub-chronic and mutagenicity studies

**DOI:** 10.1016/j.toxrep.2019.06.003

**Published:** 2019-06-07

**Authors:** Susumu Yoshino, Riyo Awa, Nobuaki Ohto, Yasuo Miyake, Hiroshige Kuwahara

**Affiliations:** Research Center, Maruzen Pharmaceuticals Co., Ltd., 1089-8 Sagata, Shinnichi, Fukuyama, Hiroshima, 729-3102, Japan

**Keywords:** 2AA, 2-aminoanthracene, AF-2, 2-(2-furyl)-3-(5-nitro-2-furyl)-acrylamide, BαP, benzo alpha-pyrene, ICR-191, 2-methoxy-6-chloro-9-[3-(2-chloroethyl)-aminopropylamino]acridine-2HCl, KP, Kaempferia parviflora, *Kaempferia parviflora*, Safety, Sub-Chronic toxicity, Mutagenicity, No-observed-adverse-effect level

## Abstract

*Kaempferia parviflora* (KP), also known as Krachai-dam in Thailand, belongs to the family Zingiberaceae and has been used traditionally to improve blood flow and treat inflammatory, allergic, and gastrointestinal disorders. The objective of this study was to investigate the safety profile of a standardized hydroalcoholic KP rhizome extract via mutagenicity and sub-chronic toxicity evaluations using *in vitro* and *in vivo* techniques. The in vitro mutagenicity of KP extract was assessed via reverse mutation tests using *Salmonella typhimurium* TA98, TA100, TA1535, and TA1537, and *Escherichia coli* WP2 *uvrA*. The sub-chronic toxicity profile was evaluated after daily oral administration of KP extract to Sprague-Dawley rats for 90 days. General toxicological parameters were monitored weekly. After the treatment period, blood was collected for hematological and biochemical analyses and certain organs were removed for macroscopic and histopathological analyses.

Reverse mutation tests revealed that KP extract did not induce gene mutations at any of the concentrations tested. In the sub-chronic toxicity test, a few changes were observed, including increased salivation in the animals administered high-dose KP extract (249 mg/kg body weight (bw)/day). No toxicologically relevant changes were observed in the biochemical analysis. Sub-chronic administration of KP extract increased platelet levels in animals administered low-dose KP extract (25 mg/kg bw/day). However, the hematological and biochemical parameters remained within normal physiological ranges for the animal species. No toxicological changes were observed in the macroscopic and histopathological analyses performed in this study. These results demonstrate that KP extract is not genotoxic and that 90-day oral administration of the doses tested did not result in toxicity. Therefore, KP extract has a high safety margin for daily use.

## Highlights

• *Kaempferia parviflora* extract exhibited no genotoxicity.
• The no-observed-adverse-effect-level (NOAEL) of the extract was > 249 mg/kg.
• Our results indicate that *Kaempferia parviflora* extract has a high degree of safety.

## 1 Introduction

Medicinal plants have been used in the prevention and cure of various diseases based on traditional knowledge for a long time. These plants are often considered safe because of the absence of adverse events. However, many of these plants have not been subjected to toxicological tests to establish their safety profiles [1,2].

*Kaempferia parviflora* Wall. ex Baker (KP), commonly known as Krachai-dam in Thailand, is a member of the Zingiberaceae family. Its rhizomes are used to treat a variety of gastrointestinal disorders [3], improve blood flow [4,5], and as a traditional treatment for inflammatory and allergic disorders [6,7]. An alcoholic extract of KP rhizomes is also used by communities in Northern Thailand to treat inflammation [8,9], as a spasmolytic [10], and to treat gastric ulcers [11]. Recently, a standardized extract of KP was reported to suppress body weight increase, body fat accumulation, and glucose intolerance in obese mice [[12], [13], [14]]. Other reports have suggested that oral intake of standardized KP extract increases energy expenditure and fat utilization [15,16]. Moreover, dietary intake of standardized KP extract reportedly decreased body fat (both visceral and subcutaneous) in overweight and pre-obese individuals [17]. Collectively, these findings suggest that KP extract could reduce body fat in humans.

KP extract has been widely used as a functional food and dietary supplement in various forms over the centuries. KP extract is considered safe as there have been no reports of adverse effects related to its use. A previous study examining daily administration of KP extract for 6 months in rats reported no toxicological effects [18]. Other studies reported that daily intake of KP extract for 12 weeks had no adverse effects on blood chemistry parameters [17,19,20]. However, the safety profiles of KP extract are still unavailable and more data are needed. This study was carried out to address this gap. Standardization of the hydroalcoholic KP extract used in this study was ensured via implementation of strict in-process controls during manufacturing. We assessed the mutagenicity and sub-chronic toxicity profiles of standardized KP extract using an *in vitro* bacterial reverse mutation test and an *in vivo* 90-day repeated oral toxicity experimental model.

## 2 Materials and methods

### 2.1 Materials

Dried KP rhizomes were received from a selected farm in Thailand. An authentic sample of KP extract (KPFORCE^™^, lot no: 61121028; Maruzen Pharmaceuticals Co., Ltd., Hiroshima, Japan), prepared via hydroethanolic extraction followed by spray drying, was obtained by implementing strict in-process manufacture control along with a detailed certificate of analysis indicating its phytochemical and nutritional compositions, microbiological status, and heavy metal content (quality assurance criteria of KPFORCE^™^ listed in Supplementary Table 1). KPFORCE^™^ contained the same amounts of dextrin,γ-cyclodextrin and KP extract (i.e. 100 mg of KP extract, 100 mg of dextrin and 100 mg of γ-cyclodextrin in 300 mg of KPFORCE^™^). KPFORCE^™^ standardized polymethoxyflavone (8.0–12.0%), and the following six compounds identified via high-performance liquid chromatography: 5,7-dimethoxyflavone, 3,5,7-trimethoxyflavone, 5,7,4′-trimethoxyflavone, 3,5,7,4′-tetramethoxyflavone, 5,7,3′,4′-tetramethoxyflavone, and 3,5,7,3′,4′-pentamethoxyflavone.

### 2.2 Genotoxicity studies (bacterial reverse mutation test)

Bacterial reverse mutation tests were conducted at the BoZo Research Center Inc. (Tokyo, Japan). The ability of KPFORCE^™^ to induce reverse mutations at the histidine loci of *Salmonella typhimurium* TA98, TA100, TA1535, and TA1537 (Division of Genetics and Mutagenesis, National Institute of Health Sciences, Japan) and *Escherichia coli* WP2 *uvrA* strains (National Institute of Technology and Evaluation, Japan) was evaluated according to standard procedures [21]. The mutagenicity of KPFORCE^™^ was assessed via a preincubation method with and without the exogenous S9 metabolism activation system (Kikkoman Biochemifa Co., Tokyo, Japan). A total of six concentrations (39.1, 78.1, 156, 313, 625, and 1250 μg/plate) were evaluated for *S. typhimurium* TA100, TA1535, and TA1537 in the absence of metabolic activation. In addition, six concentrations (156, 313, 625, 1250, 2500, and 5000 μg/plate) were evaluated for *S. typhimurium* TA98 without metabolic activation and for *S. typhimurium* TA98, TA100, TA1535, and TA1537 with metabolic activation. Five concentrations (313, 625, 1250, 2500, and 5000 μg/plate) of *E. coli* WP2 *uvrA* were evaluated, irrespective of metabolic activation, as no growth inhibition was observed in the preliminary dose selection test.

Sterile water (Otsuka Pharmaceutical Factory, Inc., Tokushima, Japan) was used as the solvent control. The following standard mutagens were used as positive controls: 2-(2-furyl)-3-(5-nitro-2-furyl)-acrylamide (AF-2, Wako Pure Chemical Industries, Ltd., Japan), sodium azide (NaN_3_, Wako Pure Chemical Industries, Ltd.), 2-methoxy-6-chloro-9-[3-(2-chloroethyl)-aminopropylamino]acridine-2HCl (ICR-191, Polysciences, Inc., Warrington, PA, USA), 2-aminoanthracene (2AA, Wako Pure Chemical Industries, Ltd.), and benzo-alpha-pyrene (BαP, Wako Pure Chemical Industries, Ltd.).

For the bacterial reverse mutation test, 0.1 mL test sample, 0.1 mL bacterial suspension, and 0.5 mL S9 mixture or phosphate buffer (pH 7.4) were added to 2.0 mL top agar containing trace amounts of histidine and biotin and maintained at 45 °C. After careful mixing, the sample was layered on a minimal glucose agar plate, which was inverted after solidification of the top agar and incubation at 37 °C for 48 h. After incubation, revertant colonies were counted. The test results were considered positive if an increase ≥ 2-fold was observed in the number of revertant colonies compared with that observed in the solvent control.

### 2.3 90-Day repeated (sub-chronic) oral toxicity study

#### 2.3.1 Animals

Healthy Sprague-Dawley rats (Crl:CD, 4 weeks old) were obtained from Charles River Laboratories Japan, Inc. (Hino Breeding Center, Shiga, Japan). After 1 week acclimation, 40 rats (weighing approximately 146–166 g for males and 128–150 g for females) were assigned randomly to four groups comprising five males and five females each. The four groups included one control group and three treatment groups administered 25, 125, and 249 mg/kg bw/day KP extract, respectively, equivalent to 75, 375, and 747 mg KPFORCE^™^/kg bw/day, respectively. According to previous reports [16,17], the recommended daily KP extract intake is equivalent to 150 mg/day KPFORCE^™^. The safety factors at the maximum, medium, and lowest doses in this 90-day study were approximately 300, 150, and 30, respectively, assuming an average human body weight of 60 kg. In this 90-day toxicity study, in order to reduce the volume administered to animals, we used KP extract with the excipients removed from KPFORCE^™^.

Rats were housed individually in stainless steel wire cages with 12-h light/dark cycles. Rats were provided radiation-sterilized solid food (FR-2; Funabashi Farm Co., Ltd., Chiba, Japan) and tap water (Kaizu City, Gifu, Japan) *ad libitum*. The temperature was maintained at 22–24 °C with 40–70% relative humidity and a ventilation frequency of 10–15 air changes/h.

#### 2.3.2 Study design

The use of animals was approved by the Ethics Committee on Animal Use (Approval no: JBS-07-ROSA-494, Center of Japan Biological Chemistry Co., Ltd.) and standard procedures described in the “Guidelines on Repeat Dose Toxicity Studies” were followed (PFSB, Notification-655, Japan) [22,23].

#### 2.3.3 General observations

General observations were recorded daily. Body weights as well as food and water consumption were recorded weekly. Blood samples were obtained at the end of the study for hematological and biochemical analyses. All animals were euthanized via intraperitoneal injection of 3% sodium pentobarbital for necropsy at the end of the study, and selected organs were removed and weighed. Histological examinations were performed on all tissues.

#### 2.3.4 Hematology and biochemistry

At the end of the 90-day treatment period, the rats were fasted for 16–18 h, anesthetized via intraperitoneal injection of 3% sodium pentobarbital solution, and blood was collected from the jugular vein. Blood samples for hematological analyses were collected in tubes containing ethylenediaminetetraacetic acid. The following parameters were assessed using an XT-2000i multi-item automated hematology analyzer (Sysmex Corp., Hyogo, Japan): red blood cell count, hemoglobin, hematocrit, platelet count, white blood cell count, mean corpuscular volume, mean corpuscular hemoglobin, mean corpuscular hemoglobin concentration, and leukocyte differential counts.

For biochemical analyses, blood was collected in tubes without anticoagulant and centrifuged to obtain serum. The following serum chemistry parameters were determined using a Hitachi 7170 automated clinical analyzer (Hitachi Ltd., Tokyo, Japan): serum alanine aminotransferase, aspartate aminotransferase, alkaline phosphatase, total protein, albumin, blood urea nitrogen, creatinine, total bilirubin, glucose, total cholesterol, and triglycerides.

#### 2.3.5 Necropsy and histopathology

All rats were sacrificed and complete necropsies was performed. The brain, pituitary gland, thyroid, thymus, lungs, heart, liver, spleen, adrenal glands, kidneys, testes, prostate, ovaries, and uterus were weighed. For the thyroid, adrenal glands, kidneys, testes, ovaries, and salivary glands, paired organs were weighed together. Organ to body weight ratios (relative weights) were also calculated. In addition, the following tissue samples were obtained and fixed in 10% neutral-buffered formalin: seminal vesicles, spinal marrow, harderian gland, mesenteric lymph nodes, submandibular lymph nodes, submandibular glands, sublingual glands, pancreas, tongue, trachea, esophagus, stomach, duodenum, jejunum, ileum, cecum, colon, rectum, bladder, epididymides, vagina, skin, skeletal muscle, sternum, and femur. The testicles and eyes were fixed in Bouin’s and Davidson’s solutions, respectively. Stored organs and tissue samples from each animal in the control and high-dose groups were embedded in paraffin, sectioned, stained with hematoxylin and eosin, and examined under an optical microscope. Macroscopic lesions observed during necropsy were also examined from each animal in the other dose groups.

#### 2.3.6 Statistical analyses

Statistical analyses were performed using StatLight^®^ software (Yukms Co., Ltd., Kanagawa, Japan). Data regarding body weight, food and water consumption, urine volume, hematological and biochemical parameters, and organ weights were compared using Bartlett’s test for homogeneous variance (*p* <  0.05). Normally distributed data were compared using one-way analysis of variance and significant differences were further evaluated using Dunnett’s test for multiple comparisons (*p* <  0.05, two-tailed). Non-normally distributed data were analyzed using the nonparametric Dunnett’s method (*p* <  0.05, two-tailed).

## 3 Results

### 3.1 Bacterial reverse mutation test

As shown in Table 1, the positive controls showed a greater number of revertant colonies than the negative control. However, KP extract treatment did not increase the number of revertants in any of the tested strains in either the absence or presence of S9 metabolic activation. A dose-dependent increase in the number of revertant colonies was observed in the case of *S. typhimurium* TA1537 with S9, although this increase was < 2-fold relative to the negative control. Similar results were obtained in further confirmation tests (data not shown). No increases in the number of revertant colonies > 2-fold relative to the negative control were observed with the other test strains and no dose-dependent changes were observed.

**Table 1:** Effects of *Kaempferia parviflora* (KP) extract (KPFORCE^™^) on bacterial reverse mutations.

Treatment	Dose (μg/plate)	TA100		TA1535		WP2 *uvrA*		TA98		TA1537	
		−S9	+S9	−S9	+S9	−S9	+S9	−S9	+S9	−S9	+S9
Negative control	0	91, 114	139, 132	9, 13	8, 13	23, 24	24, 24	19, 11	31, 26	7, 5	10, 11
KPFORCE^TM^	39.1	108, 123	－	7, 8	－	－	－	－	－	7, 7	
	78.1	93, 97	－	4, 5	－	－	－	－	－	8, 4	
	156	104, 97	165, 169	11, 6	8, 6	－	－	16, 13	41, 26	4, 5	15, 15
	313	98, 107	162, 170	5, 7	12, 11	26, 24	25, 35	20, 17	50, 34	4, 5	10, 13
	625	75, 83	152, 176	5*, 7*	10, 6	20, 15	36, 29	15, 18	36, 33	3*, 6*	14, 18
	1250	44*, 68*	161, 174	4*, 3*	8, 10	16, 17	25, 28	17, 12	33, 28	1*, 1*	18, 20
	2500	－	171, 160	－	4*, 5*	16, 15	27, 19	19*, 19*	27*, 25*	－	7*, 9*
	5000	－	95*, 110*	－	4*, 3*	18, 16	19, 15	19*, 12*	35*, 25*	－	6*, 5*
AF-2	0.01	571, 588	－	－	－	67, 62	－	－	－	－	－
	0.1	－	－	－	－	－	－	321, 327	－	－	－
NaN_3_	0.5	－	－	257, 273	－	－	－	－	－	－	－
ICR-191	1.0	－	－	－	－	－	－	－	－	1070, 1052	－
BαP	5.0	－	892, 946	－	－	－	－	－	391, 427	－	95, 82
2AA	2.0	－	－	－	305, 278	－	－	－	－	－	－
	10.0	－	－	－	－	－	739, 878	－	－	－	－

Results are shown as means from two plates as indicated by the number of revertant colonies (n = 2).

Abbreviations: TA100, *Salmonella typhimurium* TA100; TA1535, *Salmonella typhimurium* TA1535; WP2 uvrA, *Escherichia coli* WP2 uvrA; TA98, *Salmonella typhimurium* TA98; TA1537, *Salmonella typhimurium* TA1537; AF-2, 2-(2-furyl)-3-(5-nitro-2-furyl) acrylamide; ICR-191, 2-methoxy-6-chloro-9-[3-(2-chloroethyl)aminopropylamino]-acridine dihydrochloride; BαP, benzo-[α]-pyrene; 2AA, 2-aminoanthracene.

* Growth inhibition was observed.

### 3.2 90-Day repeated oral toxicity study

#### 3.2.1 General observations

No animals in any of the treatment groups died during the study. Increased salivation was observed in two male and three female rats in the medium-dose group (125 mg/kg bw/day KP extract), and in four male and four female rats in the high-dose group (249 mg/kg bw/day KP extract). However, the increased salivation was mild in severity, occurred approximately 1–3 min post dosing, and only persisted for approximately 10 min in all of the affected animals.

No significant differences between the treatment groups and the control group were observed with regard to weekly body weight, food consumption, or water consumption (Supplementary Tables 2 and 3).

#### 3.2.2 Hematological and biochemical parameters

After 90 days of KP extract administration, sporadic and generally statistically non-significant changes in hematological parameters were observed (Table 2). Platelet counts in female rats in the low-dose group (25 mg/kg bw/day KP extract) were significantly higher than those in control group rats. However, no statistically significant differences were observed in the medium-dose (125 mg/kg bw/day KP extract) or high-dose (249 mg/kg bw/day KP extract) groups, indicating the absence of any dose-dependent trend. Evaluation of the biochemical parameters shown in Table 3 revealed no significant KP extract-associated changes in animals of either sex in any dose group compared with their respective controls.

**Table 2:** Effects of KP extract on hematological parameters in rats treated orally for 90 days.

Parameter	Group (females)				Group (males)			
	Control	Low-dose	Medium-dose	High-dose	Control	Low-dose	Medium-dose	High-dose
WBC (10^9^/L)	54.5 ± 10.9	63.2 ± 12.4	42.1 ± 9.0	47.5 ± 8.6	96.5 ± 12.8	91.7 ± 17.4	97.8 ± 32.3	106.0 ± 24.1
RBC (10^12^/L)	724.4 ± 21.9	752.6 ± 30.1	708.0 ± 10.2	724.0 ± 21.6	788.6 ± 41.2	788.8 ± 32.4	791.8 ± 35.7	770.4 ± 11.5
HG (g/L)	14.3 ± 0.2	14.5 ± 0.6	14.0 ± 0.4	14.1 ± 0.2	14.7 ± 0.6	15.0 ± 0.5	14.7 ± 0.8	14.5 ± 0.2
HC (%)	39.3 ± 0.8	39.4 ± 1.1	37.9 ± 1.1	38.6 ± 0.9	40.4 ± 1.2	41.3 ± 1.2	40.3 ± 2.2	40.0 ± 0.5
PLT (10^12^/L)	82.8 ± 9.1	100.4 ± 12.5*	88.6 ± 10.3	83.3 ± 5.7	96.2 ± 3.4	93.3 ± 11.7	104.4 ± 13.7	96.7 ± 9.9
MCV (fL)	54.3 ± 1.3	52.3 ± 1.0	53.6 ± 1.6	53.4 ± 1.2	51.3 ± 1.5	52.4 ± 2.2	50.9 ± 2.3	52.0 ± 1.3
MCH (pg)	19.8 ± 0.6	19.2 ± 0.4	19.8 ± 0.6	19.5 ± 0.5	18.7 ± 0.5	19.0 ± 0.5	18.5 ± 0.8	18.8 ± 0.5
MCHC (g/dL)	36.5 ± 0.4	36.8 ± 0.6	37.0 ± 0.2	36.5 ± 0.5	36.5 ± 0.4	36.3 ± 0.6	36.4 ± 0.4	36.1 ± 0.4
Lymphocytes (%)	78.3 ± 8.5	82.4 ± 8.2	80.8 ± 6.0	85.3 ± 1.3	79.3 ± 4.0	81.9 ± 4.7	82.4 ± 2.0	83.5 ± 3.5
Neutrophils (%)	16.8 ± 8.1	12.9 ± 8.3	14.1 ± 4.9	10.0 ± 0.4	15.3 ± 3.4	12.8 ± 4.1	12.7 ± 1.3	12.1 ± 2.7
Other (%)	4.9 ± 1.1	4.7 ± 2.1	5.1 ± 1.3	4.7 ± 1.5	5.4 ± 0.7	5.3 ± 1.9	4.9 ± 1.3	4.4 ± 1.6

Results are shown as means ± standard deviation (n = 5).

Low dose: 25 mg/kg bw/day; medium dose: 125 mg/kg bw/day; high dose: 249 mg/kg bw/day.

Abbreviations: WBC, white blood cell count; RBC, red blood cell count; HG, hemoglobin; HC, hematocrit; PLT, platelet count; MCV, mean corpuscular volume; MCH, mean corpuscular hemoglobin; MCHC, mean corpuscular hemoglobin concentration.

* Statistically significant difference relative to the control (*p* <  0.05).

**Table 3:** Effects of KP extract on biochemical parameters in rats treated orally for 90 days.

Parameter	Group (females)				Group (males)			
	Control	Low-dose	Medium-dose	High-dose	Control	Low-dose	Medium-dose	High-dose
AST (U/L)	64.2 ± 2.9	69.4 ± 9.8	59.4 ± 5.4	67.4 ± 27.3	94.2 ± 24.8	71.6 ± 15.8	87.8 ± 31.8	83.2 ± 17.6
ALT (U/L)	26.6 ± 4.8	31.6 ± 5.5	26.2 ± 3.4	28.4 ± 12.1	40.4 ± 18.3	29.4 ± 5.9	34.4 ± 11.8	35.4 ± 7.2
ALP (U/L)	110.4 ± 23.2	121.6 ± 34.4	147.4 ± 45.1	139.0 ± 88.1	248.2 ± 56.3	246.2 ± 65.9	236.4 ± 13.3	291.8 ± 63.4
Total protein (g/L)	61.0 ± 4.0	61.8 ± 4.0	64.2 ± 4.8	62.6 ± 2.9	56.2 ± 1.3	57.0 ± 2.4	53.8 ± 2.8	55.4 ± 3.5
Albumin (g/L)	30.2 ± 2.8	31.0 ± 2.3	32.4 ± 3.1	31.0 ± 2.1	24.0 ± 1.0	23.8 ± 0.8	22.8 ± 1.3	24.2 ± 1.6
BUN (mmol/L)	6.44 ± 0.48	6.45 ± 1.15	5.38 ± 0.84	6.13 ± 1.25	5.79 ± 0.37	5.88 ± 0.29	6.26 ± 0.54	5.91 ± 0.58
CRE (mmol/L)	33.59 ± 7.40	33.59 ± 3.95	31.82 ± 4.84	26.52 ± 0.00	30.06 ± 4.84	26.52 ± 6.25	30.06 ± 4.84	24.75 ± 3.95
T-BIL (μmol/L)	1.0 ± 0.9	1.0 ± 0.9	1.0 ± 1.5	1.4 ± 0.8	0.7 ± 0.9	0.7 ± 0.9	0.0 ± 0.0	0.0 ± 0.0
Glucose (mmol/L)	81.38 ± 10.06	82.82 ± 9.77	81.04 ± 6.79	79.93 ± 5.30	84.71 ± 10.06	85.04 ± 13.39	80.71 ± 8.81	85.37 ± 12.35
T-Cho (mmol/L)	1.83 ± 0.33	1.69 ± 0.18	2.16 ± 0.73	2.08 ± 0.13	1.72 ± 0.27	1.83 ± 0.43	1.74 ± 0.29	2.03 ± 0.44
TG (mmol/L)	0.48 ± 0.10	0.61 ± 0.30	0.99 ± 0.55	0.74 ± 0.24	0.72 ± 0.34	0.84 ± 0.34	0.84 ± 0.61	1.03 ± 0.37

Results are shown as means ± standard deviation (n = 5).

Low dose: 25 mg/kg bw/day; medium dose: 125 mg/kg bw/day; high dose: 249 mg/kg bw/day.

Abbreviations: AST, aspartate aminotransferase; ALT, alanine aminotransferase; ALP, alkaline phosphatase; BUN, blood urea nitrogen; CRE, creatinine; T-BIL, total bilirubin; T-Cho, total cholesterol; TG, triglycerides.

#### 3.2.3 Pathology

Organ weight data are presented in Table 4. Compared with the control group, male rats in the low-dose group (25 mg/kg bw/day KP extract) exhibited significantly higher relative weights of the thymus and heart, and female rats in the high-dose group (249 mg/kg bw/day KP extract) showed significantly lower relative adrenal gland weight. However, these changes were not dose-dependent, and all were within the ranges of the testing institution’s historical records. As such, these changes were not considered to be biologically significant.

**Table 4:** Effects of KP extract on relative organ weights in rats treated orally for 90 days.

Parameter		Group (females)				Group (males)			
		Control	Low-dose	Medium-dose	High-dose	Control	Low-dose	Medium-dose	High-dose
Liver	(g/100 g)	2.55 ± 0.13	2.57 ± 0.27	2.66 ± 0.09	2.68 ± 0.13	2.88 ± 0.21	3.03 ± 0.33	2.69 ± 0.10	3.03 ± 0.25
Kidneys	(g/100 g)	0.60 ± 0.02	0.57 ± 0.05	0.62 ± 0.04	0.61 ± 0.04	0.58 ± 0.07	0.56 ± 0.07	0.59 ± 0.05	0.61 ± 0.07
Spleen	(g/100 g)	0.20 ± 0.03	0.17 ± 0.01	0.19 ± 0.02	0.17 ± 0.03	0.16 ± 0.02	0.18 ± 0.02	0.18 ± 0.04	0.17 ± 0.03
Thymus	(g/100 g)	0.13 ± 0.01	0.12 ± 0.03	0.12 ± 0.01	0.14 ± 0.03	0.08 ± 0.01	0.10 ± 0.02*	0.09 ± 0.00	0.09 ± 0.02
Heart	(g/100 g)	0.33 ± 0.05	0.35 ± 0.03	0.32 ± 0.03	0.33 ± 0.02	0.27 ± 0.03	0.32 ± 0.04*	0.27 ± 0.01	0.28 ± 0.02
Brain	(g/100 g)	0.63 ± 0.05	0.67 ± 0.06	0.60 ± 0.06	0.60 ± 0.08	0.38 ± 0.02	0.38 ± 0.03	0.36 ± 0.03	0.39 ± 0.04
Lungs	(g/100 g)	0.37 ± 0.03	0.36 ± 0.03	0.36 ± 0.02	0.34 ± 0.03	0.27 ± 0.01	0.26 ± 0.03	0.26 ± 0.02	0.27 ± 0.02
Pituitary	(mg/100 g)	5.4 ± 1.5	6.0 ± 1.2	5.8 ± 1.3	5.0 ± 1.0	2.4 ± 0.5	2.4 ± 0.9	1.8 ± 0.4	2.4 ± 0.5
Thyroid	(mg/100 g)	5.8 ± 1.0	5.9 ± 0.8	5.9 ± 1.3	5.4 ± 0.9	3.7 ± 0.6	4.2 ± 0.4	4.3 ± 0.6	4.6 ± 0.5
Adrenal gland	(mg/100 g)	25.5 ± 6.1	23.3 ± 5.7	23.3 ± 3.1	18.3 ± 2.4*	11.1 ± 2.1	10.9 ± 3.2	11.5 ± 2.9	11.0 ± 2.7
Testes	(g/100 g)	－	－	－	－	0.61 ± 0.08	0.61 ± 0.03	0.54 ± 0.03	0.60 ± 0.06
Prostate	(g/100 g)	－	－	－	－	0.25 ± 0.06	0.21 ± 0.12	0.25 ± 0.05	0.27 ± 0.10
Uterus	(g/100 g)	0.24 ± 0.04	0.24 ± 0.05	0.27 ± 0.10	0.17 ± 0.01*	－	－	－	－
Ovaries	(mg/100 g)	29.7 ± 7.1	24.3 ± 5.3	26.2 ± 6.0	25.1 ± 5.5	－	－	－	－

Results are shown as means ± standard deviation (n = 5).

Low dose: 25 mg/kg bw/day; medium dose: 125 mg/kg bw/day; high dose: 249 mg/kg bw/day.

* Statistically significant difference relative to the control (*p* <  0.05).

Necropsy and histopathological examinations were performed on rats in the control and high-dose (249 mg/kg bw/day of KP extract) groups. As shown in Supplementary Table 4, histopathological examinations showed no apparent difference with regard to the severity or frequency of findings between the two groups. During necropsy, testicular edema and small epididymal size (unilateral and right, respectively) were noted in one male animal in the low-dose group (25 mg/kg bw/day KP extract). However, as these changes were sporadic, they were considered unrelated to KP extract treatment.

## 4 Discussion

KP extract has long been used to treat gastrointestinal disorders [3], improve blood flow [4,5], and as a remedy for inflammatory and allergic disorders [6,7]. KP extract is also used in commercially prepared functional foods because of its anti-oxidative, anti-inflammatory, and anti-obesity properties [6,13,17,24]. However, few studies have provided toxicological evaluations of KP extract. A previous study examining daily administration of KP extract for 6 months in rats reported no toxicological effects [18]. Other studies reported that daily intake of KP extract for 12 weeks had no adverse effects on blood chemistry parameters 17,19,20]. Moreover, a previous toxicology report of dietary ingredients including KP extract revealed no adverse effects [25]. However, safety profiles for KP extract are still unavailable and more data are needed. In this study, therefore, we investigated the toxicity profiles of KP extract via genotoxicity and sub-chronic oral toxicity studies. The results of these studies indicate that KP extract had no significant adverse effects. These data suggest the safety of KP extract and support its potential use in functional foods and/or dietary supplements.

A previous acute toxicity study in mice reported a mean lethal dose of KP rhizomes of >13.3 g/kg [26]. Through an acute toxicity study, we determined that the mean lethal dose of KP extract used in this trial was >2000 mg/kg (data not shown). Moreover, toxicity analyses of 6 months dietary intake of 500 mg/kg bw/day KP ethanolic extract in rats indicated no adverse effects [18]. Similarly, the results of the present study revealed no genotoxicity or sub-chronic toxicity related to polymethoxyflavone-standardized KP extract treatment. The statistically significant changes in platelet count and organ weight observed in some cases were not considered toxicologically relevant, as these changes were non-severe and parameters remained within the historical control ranges of the testing laboratory and/or exhibited no clear dose-response relationships. These data suggest that the KP extract is safe; however, synegistic effects have been reported for toxic stimuli [27], thus it is necessary to conduct additional safety profile studies for various combinations in the future. Furthermore, assessing the maternal toxicity and embryo/fetal development following oral administration in pregnant animals is also necessary [28].

In summary, the results of these 90-day toxicity and genotoxicity studies suggest that KP extract is safe for dietary consumption as either a functional food or dietary supplement.

## 5 Conclusions

In the present study, KP extract did not induce gene mutations in bacteria or exhibit toxicity in male or female rats after 90 days of repeated oral administration at high dose (249 mg/kg bw/day, equivalent to 747 mg KPFORCE^™^/kg bw/day). These results indicate that KP extract is not genotoxic and that its no-observed-adverse-effect level in rats is >249 mg/kg bw/day.
